# Ultrasound 3D reconstruction of malignant masses in robotic-assisted partial nephrectomy using the PAF rail system: a comparison study

**DOI:** 10.1007/s11548-020-02149-4

**Published:** 2020-05-08

**Authors:** Chongyun Wang, Charalampos Komninos, Stephanie Andersen, Claudia D’Ettorre, George Dwyer, Efthymios Maneas, Philip Edwards, Adrien Desjardins, Agostino Stilli, Danail Stoyanov

**Affiliations:** 1 Wellcome/EPSRC Centre for Interventional and Surgical Sciences (WEISS), University College London, 43-45 Foley St., Fitzrovia, London, W1W 7EJ UK; 2 Department of Electrical and Computer Engineering, University of Patras, 26504 Rio, Patras, Greece; 3 Department of Computer Science, Stanford University, 353 Serra Mall, Stanford, CA 94305 USA

**Keywords:** 3D ultrasound, Laparoscopy, Surgical robotics, Soft robotics

## Abstract

**Purpose:**

In robotic-assisted partial nephrectomy (RAPN), the use of intraoperative ultrasound (IOUS) helps to localise and outline the tumours as well as the blood vessels within the kidney. The aim of this work is to evaluate the use of the pneumatically attachable flexible (PAF) rail system for US 3D reconstruction of malignant masses in RAPN. The PAF rail system is a novel device developed and previously presented by the authors to enable track-guided US scanning.

**Methods:**

We present a comparison study between US 3D reconstruction of masses based on: the da Vinci Surgical System kinematics, single- and stereo-camera tracking of visual markers embedded on the probe. An US-realistic kidney phantom embedding a mass is used for testing. A new design for the US probe attachment to enhance the performance of the kinematic approach is presented. A feature extraction algorithm is proposed to detect the margins of the targeted mass in US images.

**Results:**

To evaluate the performance of the investigated approaches the resulting 3D reconstructions have been compared to a CT scan of the phantom. The data collected indicates that single camera reconstruction outperformed the other approaches, reconstructing with a sub-millimetre accuracy the targeted mass.

**Conclusions:**

This work demonstrates that the PAF rail system provides a reliable platform to enable accurate US 3D reconstruction of masses in RAPN procedures. The proposed system has also the potential to be employed in other surgical procedures such as hepatectomy or laparoscopic liver resection.

## Introduction

Pre-operative medical imaging techniques like computed tomography (CT) [1] and magnetic resonance imaging (MRI) [2] are generally used during the diagnostic stage of interventional healthcare. This information is however not effectively utilised during surgery at present. To ensure the surgical removal of the targeted mass is carried out without leaving any malignant tissue behind, IOUS [3] is performed. IOUS is regarded as the most effective intraoperative localisation and outlining of malignant masses in robot-assisted minimally invasive surgical procedures like partial hepatectomy [4] and partial nephrectomy [5] and for identifying blood vessels within the organ. In the context of RAPN, a drop-in transducer is deployed through a trocar port (11–13 mm depending on the model of the probe used) inside the patient abdomen, grasped with a dedicated laparoscopic tool and swiped on the targeted kidney. IOUS provides a view of the target mass that is not affected by overlying structures such as subcutaneous fat, bowel gas, or bones [6] while also avoid continuous exposition to ionising radiation as in intraoperative fluoroscopy or dyna-CT.

According to [7], during RAPN the surgeon uses a drop-in transducer to localise the malignant mass sliding the drop-in US probe on the surface of the kidney. Swipe by swipe the surgeon marks with an electro-cautery tool the margins of resection on the kidney’s surface. The US images are amalgamated and then visualised in real-time on the display of the console of the da $\hbox {Vinci}^\circledR$ Surgical System (Intuitive Surgical Inc., Sunnyvale, CA, US). When marking the margins of resection, a portion of healthy tissue must be removed around the malignant tissue to minimise the risk of relapse. Depending on the tool configuration adopted and on the patient-specific pathology there are two possible scenarios: (1) The US probe can be used at the same time of the electro-cautery tool [8]; (2) the US probe needs to be released to hold the electro-cautery tool [9]. In the latter scenario, given that the US images are displayed in real-time, the surgeon must memorise the position of the targeted mass while switching to the electro-cautery tool. Additionally, due to the morphology of the kidney, multiple swipes and multiple organ re-positioning actions are generally required, further increasing the cognitive load and the risk of organ perforation and internal bleeding. In [10] the potential benefit of the use of real-time US overlay intraoperatively has been discussed to reduce these risks. In [11], the authors also raise the problem of the carcinogenic hazard posed by the surgical smoke produced by the electro-cautery tool.

Instead of physically outlining the margins on the surface of the kidney, the relative position of the tumour can be presented using 3D-reconstructed ultrasound images of the targeted mass overlaid on the 3D viewer of the da Vinci$^\circledR$ Surgical System. Instead of producing a series of cross-sectional US images, 3D ultrasound [12] produces a volume to reconstruct the anatomy of the targeted organ. Firstly introduced in [13], there are different approaches to 3D US including: freehand [14], generating a 3D ultrasound image by inserting a series of 2D images with the correct correspondences in desired coordinates, mechanically steered [15], implementing a mechanical steering system that sweeps a linear probe back and forth within fixed housing in certain manners, mechanically rotated [16], producing a 3D image by controlled retraction of the transducer and 2D-phased array probe [17], a specifically designed transducer that outputs a 3D volume instead of a cross-sectional image. Considering the RAPN scenario and the linear drop-in transducer used we are in the case (1). Two major challenges exist when performing 3D reconstruction based on US images in a freehand scenario. The first is to guarantee contact with the targeted surface in order to generate a high-quality ultrasound image. The second is the acquisition of accurate poses of the transducer while performing the scanning. Although the scanning process is simplified thanks to the dexterity of the robotic-assisted tool, due to the limited operation space and the slippery of the target surface, this procedure is regarded as challenging and it requires skilled clinicians to be successfully performed.

**Fig. 1:**
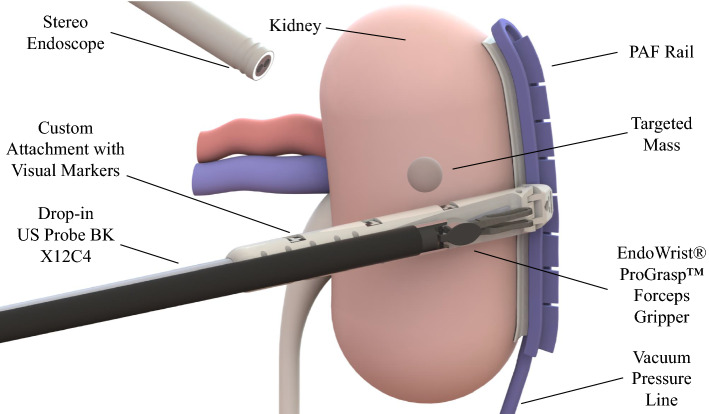
Pneumatically attachable flexible rails: overview of the system when used to guide a drop-in US probe for tumour margins outlining in RAPN procedures

In [18], the concept of pneumatically attachable flexible (PAF) rails has been proposed by the authors to enable track-guided IOUS scanning in RAPN. Illustrated in Fig. 1, the proposed system is composed of two components: the PAF rail and a customised clip-on attachment to be installed on the drop-in US transducer to enable mechanical coupling between the probe and the rail. The PAF rail is first affixed on the surface of the target organ which is the kidney in the study presented, however, the authors highlighted how the use of this system can be extended to other organs, e.g. the liver, and other procedures, e.g. partial hepatectomy. The PAF rail attaches on the organ by means of a series of bio-inspired suction cups. A vacuum pump is used to produce suction. Once the transducer is paired with the rail by means of the add-on attachment installed on it, the probe can be moved along the rail to perform the scanning sequence. By employing such system a clear and stable trajectory is defined by the rail and the pairing between the probe and the PAF rail also constrains the movement of the probe reducing its degrees of freedom (DOFs) from six to two: sliding along the rail and rotating around the attachment joint. As a result, the ultrasound probe cannot slide away from the surface and the rotational DOF is the only one that needs to be controlled to ensure contact between the probe and the surface. The resulting modified procedure is deskilled in comparison to the original one and potentially faster. Ultimately, this will enable super-imposition of the 3D reconstructed images onto the surgical images, removing the need for multiple swiping and outlining.

In this paper, we investigate how to create 3D images of the malignant masses typically targeted in RAPN based on the cross-sectional US images collected with the US probe when the PAF rail system is used. The objective of the experimental analysis presented here is to evaluate the performance of different tracking methods. The data presented have been collected using a first-generation da $\hbox {Vinci}^\circledR$ Surgical System and the da $\hbox {Vinci}^\circledR$ Research Kit (dVRK) to extract kinematics data of the patient-side manipulator (PSM) as well as videos of the stereo endoscope, building also on the work of the authors on autonomous pick-and-place of the PAF system presented in [19]. In this comparison study, we analyse the performances of three different approaches to estimate the pose of the US probe to perform 3D reconstruction of US images of a malignant mass: robot kinematics (I), visual markers embedded on the probe using single camera (II) and stereo vision (III). The resulting 3D reconstructed images of the targeted mass are then compared with a CT scan image that is used as the ground truth.

The paper is organised as follows: In section “Methods” the methods are discussed, including the hardware modifications implemented on the PAF rail system presented in [18] to improve the pairing between the grasper and the probe, as well as the modifications to embed the visual features. The proposed algorithm to segment the targeted mass is also presented. In section “Results” the overall experimental setup is presented together with the results. In section “Conclusions” the conclusions are presented.

**Fig. 2:**
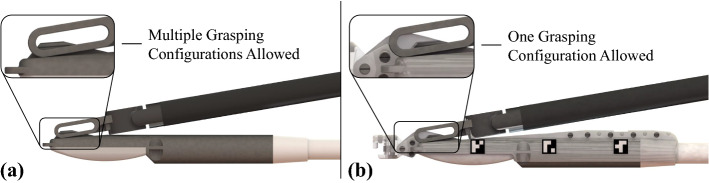
Grasping comparison: **a** grasping configuration between the $$\hbox {EndoWrist}^\circledR $$$$\hbox {Prograsp}^{\mathrm{TM}}$$ Forceps gripper and the BK X12C4 drop-in US probe in standard clinical use and **b** when the custom connector for the PAF rail system is embedded

## Methods

### Hardware modifications to the system

The work presented in this paper builds on the design presented in [18] where experiments were conducted to evaluate different designs of the PAF rail in order to maximise its adherence to the targeted organs. In this work, the optimised PAF rail design discussed in [18] is used to perform the tests described in the next section. Here a new custom attachment developed for the drop-in US probe BK X12C4 (BK-Medical Holding Inc., Peabody, Massachusetts) is introduced for the first time. The new attachment presents a number of new design features. As shown in Fig. 2, where a side-by-side comparison between the standard system and custom system is presented, the standard connector of the drop-in US probe is significantly smaller than the slot of the tool used to grasp it. This results in the grasping configuration, hence, the relative orientation between the tool and the probe, being different every time the two are paired. This makes the use of the inverse kinematic description of the manipulator unreliable for the computation of the position of the probe. With the system presented in Fig. 2b this problem does not subsist because the attachment embeds a slot that is designed to match exactly the cavities of the $$\hbox {EndoWrist}^\circledR $$$$\hbox {Prograsp}^{\mathrm{TM}}$$ Forceps, ensuring that only one grasping configuration is possible. This allows significantly more accurate computation of the probe position using the inverse kinematic description of the manipulator. Another modification is also shown in Fig. 2b: three squared slots have been added on each side of the attachment to embed visual markers. In our experiments, ArUco markers [20] are affixed into these slots.

### Pose estimation using kinematics

As introduced in section “Introduction”, kinematics data of the PSM can be recorded via the dVRK, which is the pose between the robot base and the current end-effector in the Cartesian space. Also, the scaling factors of the ultrasound image are given. In order to generate a 3D ultrasound model, we need to determine the transformation between the end-effector (*E*) and the ultrasound image plane (*P*) ${\mathbf {T}}_{\mathrm{E}}^{\mathrm{P}}$. By assuming there is a fixed grasping configuration between the end-effector and the transducer, the desired transformation is calculated as:

1 $$\begin{aligned} {\mathbf {T}}_{\mathrm{B}}^{\mathrm{U}}={\mathbf {T}}_{\mathrm{B}}^{\mathrm{E}} \cdot {\mathbf {T}}_{\mathrm{E}}^{\mathrm{P}} \cdot {\mathbf {V}}_{\mathrm{P}}^{\mathrm{U}} \end{aligned}$$

where T is 4-by-4 transformation matrices composed of a 3-by-3 rotation matrix and a 3-by-1 translation vector, ${\mathbf {T}}_{\mathrm{B}}^{\mathrm{E}}$ is the recorded PSM data presented as 4-by-4 transformation matrices, ${\mathbf {T}}_{\mathrm{E}}^{\mathrm{P}}$ is the unknown 4-by-4 transformation remained to be determined and ${\mathbf {V}}_{\mathrm{P}}^{\mathrm{U}}$ is the 4-by-1 vector containing scaling factors that transform from world coordinate system in mm to ultrasound image plane in pixel.

In order to calculate ${\mathbf {T}}_{\mathrm{E}}^{\mathrm{P}}$, a calibration experiment is conducted following the rigid-body point-based registration method detailed in [21–23]. Multiple swipes are performed using the ultrasound transducer on the surface of a tank filled with water with a silicon carbide ceramic sphere glued to the bottom, keeping the sensing surface of the probe submerged and pointed towards it. In this experiment, ${\mathbf {T}}_{\mathrm{E}}^{\mathrm{P}}$ is calculated by minimising the fiducial registration error (FRE), which can be represented as:

2 $$\begin{aligned} {\mathrm {FRE}}^{2} \equiv \frac{1}{N} \sum _{t=1}^{N}|R x+t-y|^{2} \end{aligned}$$

where N is the number of data, *x* is the 3D coordinate of fiducial points observed in one coordinate system, *y* is the 3D coordinate observed in the other coordinate system and $T = [R\ t]$ is the rigid transformation matrix that maps fiducial points from one coordinate systems to the other one. Stated in [23], the solution can be calculated using singular value decomposition (SVD) [24]. This experiment is developed based on an assumption that the connection between the end-effector and the transducer is only one grasping configuration, which, as explained in section “Hardware modifications to the system”, is not in the original design. After switching to the updated attachment, the grasping configuration is physically constrained and ${\mathbf {T}}_{\mathrm{E}}^{\mathrm{P}}$ is measured directly from the CAD model.

### Optical tracking

After switching to the updated attachment, there are three square marker slots on each side of the transducer, where visual markers are affixed. Shown in Fig. 2b, the Aruco marker is a N-by-N asymmetric rectangular matrix with a black boundary. Each matrix is designed to be unique and can be generated or decoded using specific dictionaries. Thus, we can affix multiple markers on the same side for comparison. The detection of the Aruco marker requires a series of steps: (1) Segment the original image and apply edge detection. (2) Enhance borders and extract rectangular contours. (3) Perform perspective projection and separate the targeted region into regular binary grids. (4) Generate four sets of codes corresponding to four possible rotations of targeted grids. (5) Check if any of these codes match the dictionary. As a result, we are able to know the ID of the marker together with its orientation and four corner coordinates if there is a match in the dictionary. The pose of the marker then can be estimated by solving a Perspective-n-Point (PnP) problem. Meanwhile, we also triangulate corner points by means of stereopsis visual cues from the stereo endoscope, which gives us four 3D coordinates of the targeted marker. The pose then is estimated by solving a least-square optimisation problem using SVD [25]. For both visual tracking methods, the pose we estimated is in the camera coordinate. Because the markers we used are affixed into the marker slots on the attachment, which is directly mounted onto the transducer, there are constant transformations from the marker slots to the US image plane that can be measured from the CAD model.

### Tumour segmentation and visualisation

Suffering from the speckle noise, it is difficult to segment the mass from the phantom in ultrasound images. Thus, a feature extraction algorithm is proposed to detect the region of the mass and outline its boundary, which is summarised as follows:

1. Apply a high-pass filter to the image to sharpen it;
2. Apply image thresholding to the sharpened image;
3. Find the connected components using blob analysis;
4. Apply edge detector to targeted components and find their contours;
5. Fit an ellipse to each contour in the original image.

An example of the application of feature extraction algorithm is available at the following link: https://bit.ly/2UJmEk6. In this case, the region of the mass is replaced by an ellipse with high greyscale value. To eliminate false positives in the detection, we mark certain frames that can see the targeted mass and only apply the algorithm on these frames with a predefined rectangular region. We also kept the bottom surface of the phantom in the segmented image for comparison. The 3D model is generated using the PLUS software library [26], by which segmented US images are inserted into a 3D space with their poses calculated with our approach.

**Fig. 3:**
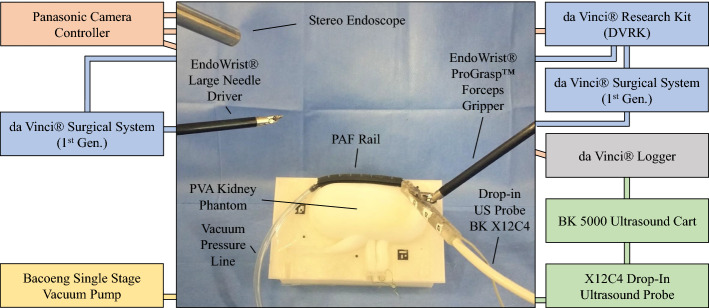
Experimental setup for phantom tests: the setup includes: the PVA kidney phantom embedding the targeted mass, the PAF rail system, the drop-in US probe BK X12C4 embedding the custom connector to enable mechanical coupling with the PAF rail system, the BK-5000 US cart to collect the US data, the da $$\hbox {Vinci}^\circledR $$ Surgical System (I Generation) fitted with the $$\hbox {EndoWrist}^\circledR $$$$\hbox {Prograsp}^{\mathrm{TM}}$$ Forceps to drive the drop-in US probe and the $$\hbox {EndoWrist}^\circledR $$ Large Needle Driver to position the PAF rail, the da $$\hbox {Vinci}^\circledR $$ Research Kit to collect kinematic and video data, the Panasonic camera controller to collect the data from the stereo endoscope and the single-stage vacuum pump to supply the vacuum pressure to the PAF rail to enable suction between the suction cup and the phantom surface

## Results

### Experimental setup

Illustrated in Fig. 3, the proposed system consists of a standard da $\hbox {Vinci}^\circledR$ surgical system, a BK5000 ultrasound machine with a X12C4 drop-in transducer (BK-Medical Holding Inc., Peabody, Massachusetts) and an updated PAF rail system. The surgical system is equipped with a forceps gripper and a stereo endoscope. The endoscope produces 576i stereo images at $\approx 30$ Hz via the dVRK, while kinematics is streamed at 60 Hz; US images are recorded at 60 Hz by the da $\hbox {Vinci}^\circledR$ logger (courtesy of Intuitive Surgical Inc., Sunnyvale, CA, US). Considering the unstable frame rate of the endoscopic images and possible frame drops, all the data are synchronised and downsampled to 25 Hz. The suction of the PAF rail system is generated by a vacuum pump, kept in a vacuum chamber and monitored with the embedded manometer. Three Aruco markers are affixed onto the add-on attachment which is mounted to the transducer to facilitate optical tracking. Tracking of the Aruco markers relies on having clear edges around the marker and the accuracy of the corner coordinates directly affect the quality of pose estimation and triangulation. Marker slots on the attachments are painted to white manually. For optical tracking, two Aruco dictionaries are tested: the standard 4-by-4 dictionary and a customised 3-by-3 dictionary. For ex-vivo experiments, US scans were performed on a custom kidney-shaped phantom that was developed with methodologies presented in [27, 28]. The phantom was designed using CAD software and its negative mould was generated. The mould was then 3D printed in polylactic acid (Ultimaker 3), and tissue-mimicking material (polyvinyl alcohol—PVA) was poured and casted within it. To simulate a malignant mass, a spherical structure was fabricated and embedded within the phantom, following the same method used to make the kidney phantom except with an additional freeze–thaw (FT) cycle [28] The additional FT cycle increased the stiffness of the mass, thereby providing more realistic mechanical properties. A radiopaque contrast agent was also added to the mass, which conferred visibility with CT imaging (O-arm surgical imaging system; Medtronic plc, Dublin, Ireland).

**Fig. 4:**
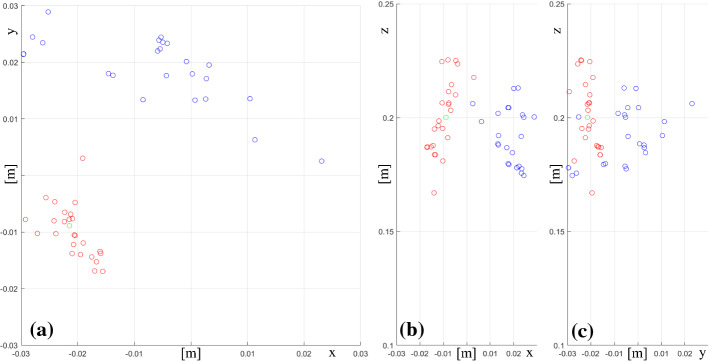
Distribution of the positions of the tip of the end-effector (ProGrasp) mounted on the PSM (in Blue) and distribution of the calculated positions of the ceramic sphere (in Red) using the optimised transformation in the *xy* (**a**), *xz* (**b**) and *yz* (**c**) planes. Green circle is the real position of the ceramic sphere

### Kinematics calibration

As detailed in section “Pose estimation using kinematics”, a calibration experiment is conducted to estimate the transformation between the PSM and the ultrasound image plane in the standard clinical scenario where the US probe is grasped with the $\hbox {Prograsp}^{\mathrm{TM}}$ Forceps gripper. We take 25 frames together with their kinematics data. Each image has a clear view of the ceramic sphere. The position of the sphere in the robot base coordinate frame is calculated based on the assumption that all these image planes intersect at the same point, which can be solved by SVD and is used as the initial value in the registration problem. After being optimised by the Levenberg-Marquardt algorithm, the minimum FRE becomes 1.00e−4, while the initial FRE is 2.77e−4. Figure  4 shows in the *xy* (a), *xz* (b) and *yz* (c) planes the position of the sphere, the positions calculated from the PSMs data and the optimised transformation. In an ideal scenario, all the calculated positions of the sphere lie right at the correct position proving that there is a fixed grasping configuration between the gripper and the transducer. However, given the multiple grasping configurations possible, the calculated positions do not coincide with the real position of the ceramic sphere, due to the unknown slippage between the gripper and the transducer. As explained in 2.2, to overcome this design limitation of the system, we introduce the custom attachment presented in Fig. 2b to allow a single grasping configuration.

### Motion estimation

To evaluate the performance of different tracking methods, experiments are conducted on the kidney phantom with the endoscope fixed in position and the phantom placed in the lower half of its mould to provide a stable platform to be clamped to the operating table. Kinematics data, endoscopic and ultrasound images were collected from multiple swipe sequences in this experiment. A swipe sequence is considered successfully completed when the probe moves from one side of the kidney to the other without losing contact with the kidney surface and the embedded mass is correctly displayed in the ultrasound images collected. Two scenarios are considered: freehand and track-guided scanning. Tracking performance are evaluated in terms of success rate (SR) (only for marker detection), average duration (AD) of the swipe sequence, average relative displacement (AveDP) between one estimated position of the probe and the subsequent one, standard deviation of displacement (StdDP), average relative rotation (AveR) of the probe between one estimated position and the subsequent one and standard deviation of rotation (StdR). Because pose estimation using kinematics is conducted based on the assumption that there is only one grasping configuration between the PSM and the transducer with the updated attachment, which is measured from its CAD model, kinematics data is considered as the ground truth. Table 1 compares the performance of the investigated methods for pose estimation of the US probe in five different scenarios: freehand based on kinematics data, 3-by-3 and 4-by-4 Aruco markers; track-guided based on kinematics data and 3-by-3 Aruco markers, comparing the data of ten swipes for each of the freehand cases and of five swipes for track-guided cases. Compared to a standard 4-by-4 Aruco marker, which is composed of 16 square binary grids, a 3-by-3 marker consists of only 9 grids. Thus, its area per grid is larger than 4-by-4’s, assuming the total area of the marker is the same. This increases significantly the success rate of marker’s detection, especially in this case, where relatively low-resolution images (576i) are used. This is reflected by the data in Table 1. In light of this, after the freehand testing, 4-by-4 markers were discarded. The kinematics of track-guided swipes, compared to that of freehand swipes, shows longer scanning duration and smaller relative displacement and rotations, owing to the physical connection between the attachment and the rail. The rail defines a trajectory and constrains the movement of the transducer, maintaining it perpendicular to the targeted surface. The slower scanning speed and the mechanical coupling between the probe and the rail reduce motion blur in endoscopic images, increasing the SR of marker detection and the reliability of pose estimation. Thus, optical tracking using 3-by-3 markers shows a higher success rate in track-guided scanning when compared to freehand case. Considering the unknown slippage within kinematics data, relative displacement and rotation of single-camera tracking lie within reasonable tolerances. We also tested pose estimation using stereo-camera tracking. Because the performance of both single-camera and stereo-camera tracking relies on accurate corner coordinates, the corner optimisation algorithm AprilTag 2 described in [29] is implemented to refine marker detection.

**Table 1:** Quantitative evaluation of the accuracy of the pose estimation of the US probe in freehand scanning and in track-guided scanning (when the attachment is paired with the PAF rail system)

	SR (%)	AD (s)	Displacement (mm)	Rotation ($^{\circ }$)
Freehand kinematics	NA	5.4	$3.62\pm 2.97$	$20.33\pm 17.82$
Freehand 3-by-3	79.9	5.4	$4.77\pm 5.29$	$25.29\pm 23.67$
Freehand 4-by-4	48.7	5.4	$5.73\pm 8.12$	$35.65\pm 26.26$
Track-guided kinematics	NA	13.3	$0.24\pm 0.16$	$0.12\pm 0.22$
Track-guided 3-by-3	83.5	13.3	$2.89\pm 4.67$	$3.22\pm 2.60$

The pose estimation data of the US probe are compared when computed from kinematics data, from tracking $3\times 3$ Aruco markers and from tracking data of $4\times 4$ Aruco markers. Displacement unit in mm and values shown in format AveDP ± StdDP. Rotation unit in degrees and values shown in format Ave*R* ± Std*R*

As shown in Table 2, this approach manages to increase the SR of both tracking methods to a satisfying level and also helps to reduce random errors in optical tracking. Compared to single-camera tracking, the stereo method produces much smaller relative displacement, which is even smaller than kinematics, as shown in Table 2. Considering displacement is the average of four 3D points triangulated by stereopsis, its precision depends on the resolution of stereo images. Thus, both tiny motions and small random errors may not be reflected. Additionally, due to the insufficient number of input for least-square optimisation, relative rotation of stereo-camera tracking is a lot worse than those of single-camera tracking and robot kinematics. Whereas the relative rotation of single-camera tracking is further optimised by the AprilTag 2 approach. Thus, we replace the rotation from stereo-camera estimation with that from single-camera estimation while keeping the displacement. However, the translation vector obtained via stereo triangulation is also calculated simply by taking the average of four data, which, similar to the rotation matrix, is likely to be affected by random errors due to the insufficient number of key points.

**Table 2:** Performance of the single and stereo camera optical tracking methods for the detection of $3\times 3$ Aruco markers in track-guided swipes with and without the AprilTag 2 corner optimisation algorithm

	SR (%)	Displacement (mm)	Rotation ($^{\circ }$)
Single without the AprilTag 2	86.2	$2.89\pm 4.67$	$3.22\pm 2.60$
Stereo without the AprilTag 2	83.3	$0.10\pm 0.09$	$57.08\pm 47.65$
Single with the AprilTag 2	99.2	$0.40\pm 0.37$	$2.35\pm 1.41$
Stereo with the AprilTag 2	97.77	$0.08\pm 0.08$	$16.10\pm 15.85$

Displacement unit in mm and values shown in format $AveDP\pm StdDP$. Rotation unit in degree and values shown in format Ave$R\pm$ Std*R*

**Fig. 5:**
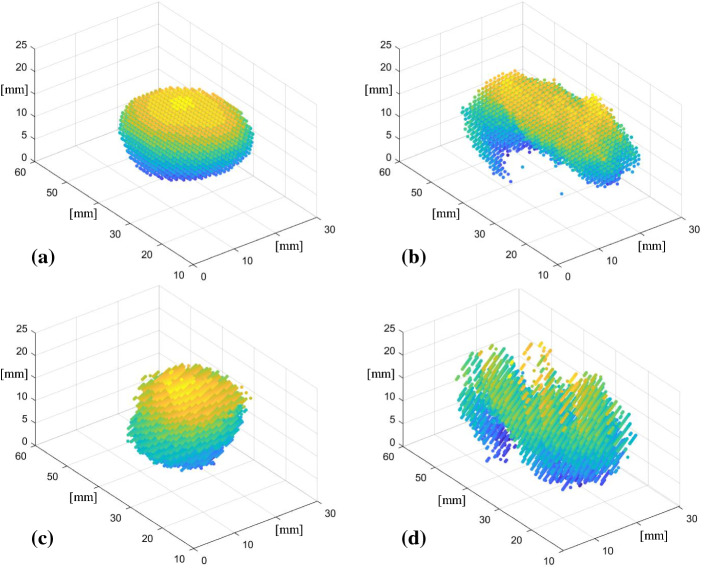
Meshes of the targeted mass inside the kidney phantom plotted as point clouds built using CT data (**a**), ultrasound data based on DVRK kinematics data (**b**), single- (**c**) and stereo-camera (**d**) data based on visual markers tracking

### Model evaluation

To evaluate the performance of different tracking methods in terms of tumour reconstruction, ex vivo experiments are conducted on the phantom. For each swipe, we record kinematics data, stereo endoscopic images and ultrasound images. An example of the stereo endoscopic and ultrasound images collected during a full swipe sequence is available at the following link: https://bit.ly/38i0GJ0. Then, three tumour meshes are generated using different methods. CT images of the phantom in the lower half of its mould are available at the following link: https://bit.ly/2T6eIHd together with a reconstructed image where it can be seen that the mass lies in the middle of the phantom, away from the bottom surface. Because also during ultrasound scanning the phantom is placed in the lower half of its mould to improve its stability and because the mould has a higher density, than the phantom itself, the surface in the generated meshes is likely to be the surface of the mould instead of the surface of the phantom. Also, because the phantom was scanned together with the lower half of its mould it is difficult to separate these two surfaces from the CT model. To evaluate the reliability of different tracking methods, we first perform 3D reconstruction of the targeted mass using the feature extraction algorithm described in 2.4 using kinematics, single-camera and stereo-camera to estimate the position of the probe. An example of data set of segmented images collected during a complete swipe sequence and used to perform the 3D reconstruction is available at the following link: https://bit.ly/350PjEB. We then extract meshes from the generated models (Fig. 5b–d) and compare with the mesh of the targeted mass obtained from the CT model (Fig. 5a) using iterative closest point (ICP) algorithm. The data were analysed with an Intel Core i7-9750H CPU and to go from raw US images to segmented images to meshes it took: 10 s for the kinematics case, 30 s for single-camera tracking and 1 min and 20 s for stereo-camera tracking. As shown in Table 3, the best mesh is achieved using single-camera tracking. As previously stated, the much lower relative displacement from stereo-camera tracking results in a higher root mean square error (RMSE) value. The stereo method suffers from the insufficient number of key points obtained using Aruco markers. Meanwhile, the quality of stereo calibration remains to be further evaluated, which may contribute to the high RMSE as well. Additionally, our feature extraction algorithm is developed based on blob analysis, which only works when the tumour area exceeded the predetermined minimum. Hence, this fails to be detected properly in some slices.

**Table 3:** RMSE values of the distances between the matched pairs of points composing the meshes of the US reconstructed model of the targeted mass based on the data of three different modalities analysed (kinematics data, single-camera and stereo-camera) and the CT model

	RMSE (mm)
Kinematics	1.63
Single-camera tracking	0.76
Stereo-camera tracking	2.26

## Conclusions

In this paper, we have experimentally investigated the feasibility of using the PAF rails [18] to assist 3D US reconstruction of malignant masses. Multiple pose estimation methods were investigated to localise the 2D US slice in the 3D space, including robot kinematics, single- and stereo-camera tracking and we compared the performance of tracking methods with different configurations. Quantitative results of 3D models from ex-vivo experiments demonstrate that the model generated using single-camera tracking has the minimum RMSE (0.76 mm) compared to the ground truth from CT scans. Future work will focus on improving the 3D visualisation of the targeted mass, optimising current tracking methods and integrating new ones, analysing organs deformations during ultrasound scans and developing an adaptive feature extraction algorithm. A wider comparison study where standard US optical reconstruction is performed will be also considered for future work, together with the use of electro-magnetic markers to finely track the motion of the probe. Given that our long-term goal is to enable overlay of the 3D US reconstructed image onto the surgical video to improve visualisation, further user studies are needed to better understand clinical needs in terms of system specifications and user experience.
